# Diagnostic and therapeutic challenges in anti-NMDA receptor encephalitis associated with an ovarian immature teratoma

**DOI:** 10.1186/s12883-026-04918-1

**Published:** 2026-05-01

**Authors:** Mahmoud Doudein, Sherehan M. Doudin, Raghad A. Lahlouh, Ola Omarya, Ahmed Ismail

**Affiliations:** 1 Internal Medicine Resident, Department of Internal Medicine, Istishari Arab Hospital, Ramallah, Palestine; 2 Clinical Pathologist, Department of Pathology, Istishari Arab Hospital, Ramallah, Palestine; 3 Pulmonary and Critical Care SpecialistDepartment of Critical Care, Istishari Arab Hospital, Ramallah, Palestine

**Keywords:** Autoimmune encephalitis, Anti-NMDA receptor encephalitis, Ovarian teratoma, Immature teratoma, Paraneoplastic neurologic disorder

## Abstract

**Background:**

Anti–N-methyl-D-aspartate receptor (anti-NMDAR) encephalitis is an autoimmune disorder characterized by antibodies against the NR1 subunit of the NMDA receptor. It predominantly affects young women and is frequently associated with ovarian teratomas. Early recognition, tumor removal, and immunotherapy are critical for favorable outcomes.

**Case presentation:**

We report a 33-year-old woman with subacute headache, low-grade fever, generalized weakness, and mood disturbances, who developed generalized tonic–clonic seizures and cognitive impairment. Brain MRI and CSF were initially normal, and EEG showed diffuse slowing without epileptiform discharges. Pelvic imaging revealed a large right ovarian mass. She was treated with corticosteroids, plasmapheresis, and ultimately rituximab, alongside right salpingo-oophorectomy. Histopathology confirmed an immature teratoma. Anti-NMDAR antibodies were later detected in CSF, confirming the diagnosis. Following tumor removal and immunotherapy, she achieved complete neurological recovery and remained seizure-free at six months.

**Conclusion:**

This case highlights the diagnostic challenges of anti-NMDAR encephalitis, particularly when MRI and CSF are normal. Early tumor detection and removal, combined with prompt escalation to second-line immunotherapy when needed, are essential for favorable outcomes. Immature ovarian teratomas, although rare, can trigger severe disease and warrant careful evaluation in adult women presenting with acute neuropsychiatric symptoms.

## Introduction

Limbic encephalitis is an autoimmune inflammatory disorder of the limbic system, mediated by antibodies such as anti–N-methyl-D-aspartate receptor (anti-NMDAR) or anti–leucine-rich glioma-inactivated 1 (anti-LGI1) [1]. In some cases, the autoimmune response is triggered by an underlying tumor, most commonly an ovarian teratoma, and is therefore considered paraneoplastic. Paraneoplastic cases are often associated with malignancy and may have a less favorable prognosis, whereas autoimmune forms without tumors generally respond well to immunotherapy [1, 2].

Anti-NMDAR encephalitis primarily affects children and young adults, with a strong female predominance (female-to-male ratio 4:1). Up to 58% of affected women have an associated ovarian teratoma, most of which are mature cystic teratomas [3]. In contrast, immature ovarian teratomas are rare, representing a particularly novel association [4, 5]. Importantly, immature teratomas differ from mature teratomas in oncologic management, as they often require adjuvant chemotherapy following surgical resection, based on tumor staging.

Diagnosis relies on clinical suspicion supported by neuroimaging, electroencephalography (EEG), cerebrospinal fluid (CSF) analysis, and detection of autoantibodies in serum or CSF [2]. Autoimmune encephalitis often begins with nonspecific prodromal symptoms—including fever, malaise, and headache—followed by acute neurological manifestations such as seizures, confusion, and psychiatric disturbances including agitation, psychosis (hallucinations and delusions), and mood disturbances such as acute depression or mania, along with behavioral disinhibition. Neuropsychiatric symptoms in autoimmune encephalitis are typically abrupt in onset and differ from psychiatric disorders without an identifiable underlying neurological or medical etiology, particularly given their rapid progression and association with neurological features such as seizures and altered consciousness [1].

Once autoimmune encephalitis is suspected, early tumor screening is particularly important for identifying teratomas, which, though rare, represent a critical and treatable cause [6, 7]. Pelvic ultrasound, MRI, or whole-body CT can identify an ovarian teratoma, which is present in over half of adult women with anti-NMDAR encephalitis [2].

We report a challenging case of anti-NMDAR encephalitis associated with an immature ovarian teratoma, emphasizing the diagnostic pitfalls, therapeutic strategy, and the importance of early tumor detection to highlight the novelty of this rare tumor association.

## Case presentation

### Clinical presentation

A 33-year-old previously healthy woman with no significant medical or psychiatric history presented with a subacute onset of headache, low-grade fever, generalized weakness, and behavioral changes characterized by social avoidance, anhedonia, decreased reactivity to her surroundings, and sleep disturbances, accompanied by auditory hallucinations. Within days of symptom onset, she developed a generalized tonic–clonic seizure associated with frothing at the mouth. There was no history of substance use, recent infection, or prior neuropsychiatric illness.

She was initially evaluated by an outside neurologist She was initially treated with multiple antiseizure medications for recurrent generalized tonic–clonic seizures. Despite this regimen, she continued to experience recurrent seizures in addition to progressive cognitive decline, manifesting as impaired attention and concentration, short-term memory loss, slowed processing, and difficulty following conversations, prompting admission to our tertiary care center for further evaluation.

On admission, the patient was hemodynamically stable and afebrile. Neurological examination revealed global aphasia, cognitive impairment (MoCA score 18/30) indicating moderate cognitive dysfunction in which attention, executive function, eye contact, and responsiveness affected. Cranial nerve examination was unremarkable. Motor strength was preserved, deep tendon reflexes were normal, and plantar responses were bilaterally flexor. There were no signs of meningeal irritation. Her antiseizure therapy was subsequently intensified to achieve better seizure control.

### Investigations

Brain magnetic resonance imaging (MRI) showed no focal lesions or abnormalities on T2-weighted or FLAIR sequences. Cerebrospinal fluid (CSF) analysis demonstrated normal opening pressure, normal white blood cell count, and normal protein and glucose levels. Gram stain and bacterial cultures were negative (See Table 1). Electroencephalography (EEG) revealed diffuse background slowing without epileptiform discharges and no evidence of extreme delta brush, consistent with an encephalopathic process. Empiric antimicrobial therapy was discontinued after infectious etiologies were excluded.

**Table 1:** CSF analysis done twice for the patient due to high suspicion of microbial encephalitis at time of diagnosis

Appearance	On admission	One week after admission
	Clear	Clear
RBC	8 Cell/mm	530 Cell/mm
WBC	5 Cell/mm	2 Cell/mm
Protein	15 mg/dL	10.5 mg/dL
Glucose	67 mg/dL	75 mg/dL
Culture	Negative	Negative

**Fig. 1:**
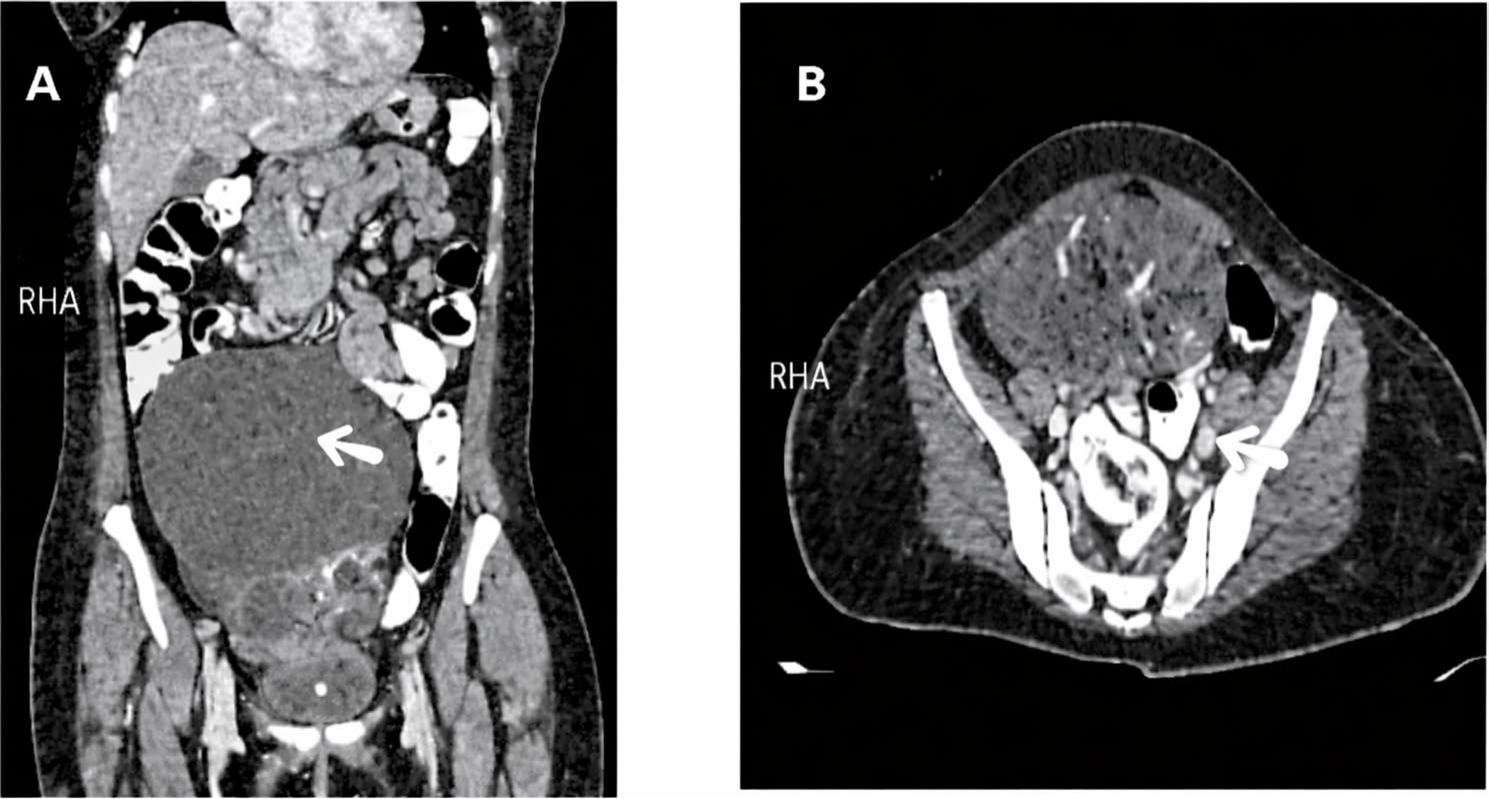
CT scan with IV contrast, image (**A**) showing coronal view, Image (**B**) showing cross sectional view (pre-op) showed solid and cystic and components of patient teratoma

Given the absence of structural, metabolic, or infectious causes, autoimmune encephalitis was strongly suspected. CSF and serum samples were sent for autoimmune and paraneoplastic antibody panels, and tumor marker evaluation was initiated. Contrast-enhanced computed tomography (CT) of the abdomen and pelvis revealed a large right ovarian mass measuring 18 × 9.4 × 17.5 cm. The lesion was septated and cystic, containing areas of fat and calcification, radiologically suggestive of an ovarian teratoma (See Fig. 1).

### Treatment and hospital course

High-dose intravenous methylprednisolone (1 g daily for 5 consecutive days) was initiated on hospital day 3. Due to minimal clinical improvement, therapeutic plasmapheresis was commenced on day 10. During her hospital course, the patient developed episodes of severe agitation, autonomic instability, and required short-term endotracheal intubation for airway protection.

Behavioral symptoms, including agitation, hallucinations, and mood disturbances, were managed with short-term psychotropic therapy. She received haloperidol 5 mg once daily for 2 days, risperidone 1 mg twice daily for 10 days, quetiapine 200 mg twice daily for 7 days, and clonazepam 1 mg three times daily for 5 days. These medications were gradually tapered over one week as her mental status improved.

**Fig. 2:**
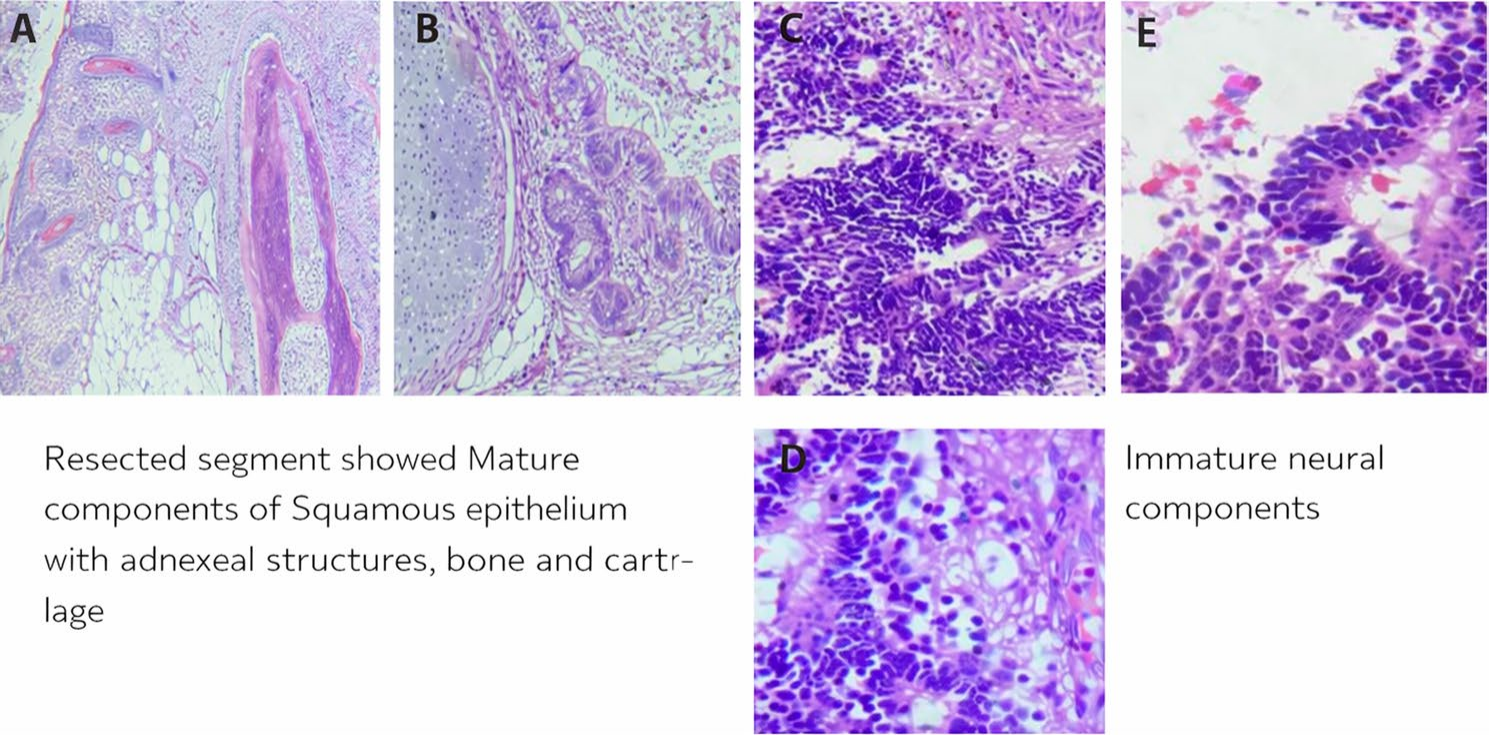
Representative histology of immature teratoma. In image (**A**) resected segment showed Mature components of Squamous epithelium with adnexeal structures, bone and cartilage, Image (**B**) showing Respiratory and colonic epithelium with cartilage. Image (**C**) showed Immature neuroepithelium components with rossete like formation. Image (**D**) represents Immature neuroepithelium components with rossete formation. Image (**E**) showed Immature neural components

In view of the ovarian mass, the patient underwent right salpingo-oophorectomy. Histopathological examination confirmed an immature ovarian teratoma (FIGO stage IA, grade II) (See Fig. 2). Tumor markers were evaluated prior to surgery and are summarized in Table 2, showing CA-125 14.5 U/mL, CA-19-9 17.1 U/mL, CA-15-3 5.10 U/mL, and AFP 0.982 ng/mL (summarized in Table 2), all within normal limits. These results indicate that tumor markers were not elevated despite the presence of a malignant ovarian teratoma, underscoring that normal tumor marker levels do not exclude immature teratomas and highlighting the importance of imaging for diagnosis. Given the malignant potential and risk of recurrence of immature teratomas, adjuvant chemotherapy with the EP regimen (etoposide and cisplatin) was initiated, while bleomycin was omitted due to inability to perform pulmonary function testing (PFT) safely. By contrast, mature teratomas typically require surgery alone without chemotherapy.

**Table 2:** Tumor markers results and interpretation

Tumor markers	Result	Interpretation
CA-125	14.5	Negative
CA-19-9	17.1	Negative
CA-15-3	5.10	Negative
AFP	0.982	Negative

Following tumor resection, the patient demonstrated gradual neurological improvement, including increased alertness, reduced agitation, and partial recovery of spontaneous speech. However, she experienced a transient clinical deterioration, characterized by worsening confusion, agitation, and decreased responsiveness. This temporary worsening is likely multifactorial, reflecting features commonly seen in anti-NMDAR encephalitis, including fluctuating disease activity, delayed response to immunotherapy. This prompted resumption of plasmapheresis and initiation of rituximab therapy (375 mg/m², administered in two doses two weeks apart), resulting in marked and rapid improvement in cognitive function, orientation, and language abilities.

CSF testing returned positive for anti–N-methyl-D-aspartate receptor (anti-NMDAR) antibodies, confirming the diagnosis. Serum testing was also positive. Antibody titers were measured using standardized cell-based assays (CBA), with CSF titer 1:46 and serum titer 1:32. While the teratoma tissue was not specifically tested for NMDA receptor expression, histopathology demonstrated mature neural tissue within the immature teratoma, which is known to potentially harbor NMDA receptor antigens.

By the time of discharge, the patient was fully oriented, seizure-free, and independent in activities of daily living. At six-month follow-up, she remained neurologically stable without seizure recurrence or neuropsychiatric relapse.

This case demonstrated a relatively rapid recovery compared to the typical protracted course of anti-NMDAR encephalitis, which often takes weeks to months for substantial neurological improvement. The early identification and resection of the immature teratoma, combined with immunotherapy, likely contributed to her favorable outcome.

## Discussion

Anti–N-methyl-D-aspartate receptor (anti-NMDAR) encephalitis is an autoimmune disorder characterized by antibodies against the NR1 subunit of the NMDA receptor, leading to a constellation of neuropsychiatric symptoms, seizures, and autonomic dysfunction [2, 3] The disorder predominantly affects children and young adults, with a strong female predominance, and is frequently associated with ovarian teratomas [3, 8].

Our patient presented with the prototypical features of anti-NMDAR encephalitis, including acute-onset seizures, cognitive impairment, behavioral disturbances, and transient aphasia. Diagnosis was challenging due to normal brain MRI, which occurs in up to 30–70% of cases [3], and unremarkable cerebrospinal fluid (CSF) findings, highlighting a well-recognized limitation: imaging and routine CSF studies can be normal in a substantial proportion of patients.

EEG findings in anti-NMDAR encephalitis are variable. In our patient, EEG revealed diffuse background slowing without epileptiform discharges, and extreme delta brush was not observed, which aligns with reports that this characteristic pattern occurs in only 30–33% of adult cases [5] While extreme delta brush can support the diagnosis, its absence does not exclude anti-NMDAR encephalitis. This underscores the importance of clinical vigilance and antibody testing, particularly when MRI and routine CSF studies are normal [3]. 

The detection of a large right ovarian mass was crucial in establishing the paraneoplastic nature of her illness. Ovarian teratomas are identified in over half of adult women with anti-NMDAR encephalitis, most commonly as mature cystic teratomas [2, 3]. Our case was notable for an immature ovarian teratoma, a rare entity that may harbor a higher antigenic load due to the presence of primitive neural tissue, potentially explaining the patient’s severe presentation and delayed response to first-line therapy [6, 9]. Unlike mature teratomas, immature teratomas require adjuvant chemotherapy according to staging guidelines, though our patient received an EP regimen (etoposide and cisplatin) without bleomycin because pulmonary function testing could not be performed [5, 10]. While chemotherapy is primarily directed at tumor eradication, literature suggests it may also reduce ongoing antigenic stimulation that could exacerbate autoimmune encephalitis, though evidence for direct symptomatic improvement is limited [5, 10].

Management of anti-NMDAR encephalitis relies on a combination of tumor removal and immunotherapy. In our patient, neurological recovery improved after tumor resection, supporting evidence that early tumor resection helps patients recover faster and lowers the risk of relapse [7, 11]. First-line immunotherapy typically consists of corticosteroids, intravenous immunoglobulin, or plasma exchange [2, 3]. Patients with incomplete response may require second-line therapies such as rituximab or cyclophosphamide, which are associated with improved outcomes and reduced relapse risk [2, 3]. In our patient, escalation to rituximab following incomplete response to corticosteroids and plasmapheresis was associated with rapid neurological recovery, which is unusual compared to typical adult cases, where recovery is often protracted over weeks to months [11].

Behavioral and psychiatric manifestations in anti-NMDAR encephalitis are commonly managed with short-term use of antipsychotics and benzodiazepines. In our patient, these agents were initiated at low doses to control agitation and psychotic symptoms, followed by gradual tapering over one week in parallel with improvement in mental status. This aligns with prior reports, where low- to moderate-dose antipsychotics and benzodiazepines are typically employed for agitation, psychosis, or catatonia [6, 12]. However, antipsychotics are purely symptomatic and do not alter the underlying disease, with potential adverse effects including extrapyramidal symptoms or neuroleptic malignant syndrome [12]. Careful dosing, short duration, and tapering alongside immunotherapy and tumor resection are recommended to safely manage psychiatric manifestations.

A transient clinical deterioration, characterized by worsening confusion, agitation, and decreased responsiveness, occurred in our patient after initial tumor resection. This is consistent with the natural fluctuations seen in anti-NMDAR encephalitis, possible delayed effects of immunotherapy, and ICU-related factors such as sedation or metabolic disturbances [3, 5] Resumption of plasmapheresis and initiation of rituximab resulted in marked and rapid improvement in cognitive function, orientation, and language abilities, highlighting the importance of vigilant monitoring and timely escalation of therapy.

Prognosis is generally favorable when early tumor removal is combined with prompt immunotherapy, with approximately 80% of patients achieving substantial recovery [3, 9]. Delayed diagnosis or failure to identify and remove the tumor is associated with poorer outcomes, prolonged hospitalization, and increased risk of relapse, which occurs in 12–25% of cases, particularly when immunotherapy is incomplete or the tumor remains unresected [3, 9]. Our patient remained neurologically stable and seizure-free at six months follow-up, illustrating the importance of timely recognition, aggressive immunotherapy, and early tumor management.

This case underscores several key clinical lessons: the need for high clinical suspicion of autoimmune encephalitis despite normal imaging and CSF, the importance of comprehensive tumor screening in adult women presenting with new-onset neuropsychiatric symptoms, the necessity of second-line immunotherapy in severe or refractory disease, and that immature ovarian teratomas, though uncommon, can serve as a potent antigenic source, reinforcing the value of individualized, multidisciplinary management strategies. Additionally, the rapid recovery observed in our patient was unusual compared to typical cases [3, 7], highlighting the variability of disease course and the potential benefit of prompt tumor resection combined with second-line immunotherapy.

## Data Availability

All data generated during this case study are included in this published article. Additional clinical details are available from the corresponding author upon reasonable request.

## Abbreviations

Anti-NMDAR: Anti–N-methyl-D-aspartate receptor
NMDA: N-methyl-D-aspartate
NR1: N-methyl-D-aspartate receptor subunit 1
MRI: Magnetic resonance imaging
FLAIR: Fluid-attenuated inversion recovery
CSF: Cerebrospinal fluid
EEG: Electroencephalography
CT: Computed tomography
FIGO: International Federation of Gynecology and Obstetrics
EP regimen: Etoposide and cisplatin
IRB: Institutional Review Board
